# Parental Perception of Changes in Basic Life Needs of Children with Disabilities after Six Months of Therapeutic Horseback Riding: A Qualitative Study

**DOI:** 10.3390/ijerph17041213

**Published:** 2020-02-13

**Authors:** Robert Lovrić, Nikolina Farčić, Štefica Mikšić, Aleksandra Gvozdanović Debeljak

**Affiliations:** 1 Faculty of Dental Medicine and Health Osijek, Josip Juraj Strossmayer University of Osijek, Osijek 31 000, Croatia; nfarcic@mefos.hr (N.F.); smiksic@fdmz.hr (Š.M.); 2 Department of Surgery, University Hospital Centre Osijek, 31 000 Osijek, Croatia; 3 Faculty of Medicine, Josip Juraj Strossmayer University of Osijek, Osijek 31 000, Croatia; 4 Elementary School Ljudevit Gaj Osijek, Osijek 31 000, Croatia; agdebeljak@mefos.hr; 5 Faculty of Education and Rehabilitation Sciences, University of Zagreb, Zagreb 10 000, Croatia

**Keywords:** children with disabilities, horseback riding, equine-assisted therapy, parent–child relations, basic life needs, quality of life

## Abstract

Therapeutic horseback riding (THR) has a positive effect on the physical, cognitive, and psychosocial functioning of children with disabilities. Parents’ reports of the effects of THR on their children support professionals in individualizing the THR program. With this qualitative study, we aimed to explore parents’ perceptions of changes in the basic life needs of their children with disabilities after six months of THR lessons and to survey parents’ explanations for the causes of these changes. The study involved parents of 13 children with disabilities who were enrolled in a six-month THR program. Parents continuously monitored their children and wrote a report on possible changes in their child’s needs according to Virginia Henderson’s need theory. Qualitative content analysis of parents’ reports indicated only positive changes in 11 children. Most codes were identified in categories “relationships and communication with other people” and “movement and posturing”. Other categories identified codes such as easier breathing, a better quality of sleep, better appetite, better elimination of stool and urine, more independence in clothing and maintaining personal hygiene, and greater interest in play and learning. Parents’ reports are further supported by the assessments of professionals. Most parents think THR is responsible for the noticeable improvements in their children’s quality of life.

## 1. Introduction

Therapeutic horseback riding (THR) is a form of mounted equine-assisted therapy that focuses on the physical, cognitive, behavioral, and psychosocial needs of the rider [1]. The use of THR is mentioned in ancient Greece, when riding was recommended to raise the spirits in chronic patients [1]. During the 1950s and 1960s, THR became a widespread practice in Europe, and in the late 1960s, in the United States. Since the late 2000s, the use of THR in children with disabilities has dramatically increased [2].

During THR, a therapeutic effect is induced on the child [3] when direct contact with the horse’s skin, horse’s body temperature, rhythmic movements of the animal, contact with therapists and other children, and the natural environment and various sensory stimuli positively affect the physical [1,4,5,6], cognitive [4,7,8], and psychosocial [2,9,10,11,12,13] functioning of children with disabilities.

A comprehensive assessment of the effects of THR on children with disabilities must combine a multidisciplinary approach (psychologists, pediatricians, speech therapists, physiatrists, physical therapists, occupational therapists, THR instructors, etc.) with the observations of disabled child’s parents. Parents are almost constantly with their child, they know their needs best, and may notice the slightest changes [4]. Thus, parental perceptions of the impact of THR on the child’s basic life needs are important and support professionals in developing individualized THR programs [14], which significantly enhance the therapeutic impact of THR on children with health issues [5]. The many benefits of THR have been described following the experiences of parents in several studies [15]. For example, in one U.S. study, parents reported significant motor, psycho-emotional, and social advances in their children with autism spectrum disorder (ASD) after THR sessions [10]. Furthermore, parents described a significant increase in their children’s confidence and self-esteem. Similarly, in one later U.S. qualitative study, Miller and Alston [16] described, through perceptions of included parents, improvements in the social and academic development of children with disabilities. The positive effects of THR on self-confidence, self-esteem, and self-worth of children with mild to moderate physical and mental disabilities were noted by parents in a study by Elliot et al. [17]. Similar experiences were also described in an Australian study in which parents of children with cerebral palsy reported that THR had a positive effect on their children’s confidence and fear of heights [18]. In a qualitative study, Stickney [1] described the positive experiences of parents who, after THR activities involving their children with ASD, noted significant improvements in their fine and gross motor skills, flexibility, and coordination of hands, as well as increased levels of empathy and confidence. The significant benefits of THR were also described in a Norwegian study by parents of children with attention deficit hyperactivity disorder (ADHD), who reported a higher level of independence, responsibility, and pride in their children [13]. In a South African qualitative study [4], parents of children with various disabilities reported significant improvements in their posture, walking, balance after THR, and in their ability to control emotions and behavior, which was accompanied with noticeable higher self-esteem and self-confidence. Similarly, in a second south African study conducted in 2017, parents observed many benefits of THR in children with ASD, such as improved verbal and non-verbal communication, increased levels of self-esteem, confidence, independence, positive mood, happiness, and satisfaction, lower anxiety level, and better emotional self-control of the child [9]. THR has a secondary positive effect on parents [4,10], which often leads to an improvement in the general quality of family life [1].

The previously mentioned studies indicate the effects of THR on children with disabilities through their parents’ perceptions. Unfortunately, the available literature does not yet offer a sufficient number of such studies [4,9], which may affect the global understanding of the role of parents as assessors of THR effects. Review of the related literature found no currently available relevant study that describes parental perceptions of the effects of THR on children with disabilities from the aspect of historically significant need theory, developed by nursing theorist Henderson [19]. Therefore, the purpose of this study was to help fill this gap by providing the results of a six-month period of parental perceptions of the effects of THR on basic life needs of their children with disabilities, following Henderson’s need theory.

Thus, we expand upon the existing global body of knowledge about THR and contribute to better understanding of how THR affects the basic life needs of children with disabilities. In particular, we sought to address the following research questions: (1) how parents perceive changes in the basic life needs of their children with disabilities after six-month THR lessons and (2) parents’ explanations for the causes of changes that have occurred in their children’s lives.

## 2. Materials and Methods

### 2.1. Theoretical Framework

The conceptual framework for this study is based on human basic life needs and has a fundamental source in Need Theory [20]. This theory is partly derived from Maslow’s hierarchy of need theory of motivation but focuses primarily on health care and assistance to people who have difficulty meeting their basic life needs, so they could be more independent as soon as possible [19]. Hence, Henderson proposed 14 components of basic life needs to be required for effective health care (Table 1) [21].

### 2.2. Study Design

This six-month qualitative study was conducted in Croatia in 2019 during implementation of a THR lesson plan as a part of European Union (EU) project entitled Developing Socially Beneficial Learning Through Equine-Assisted Therapy (ID UP.04.2.1.02.0186.). Research activities were conducted at the Croatian partner institutions involved in the project (Equestrian Club and Association for Parents of Children with Disabilities from Vukovar). One of the partners was the Faculty of Dental Medicine and Health Osijek, and the task of its nursing BSc students was to implement a modern form of socially useful learning. Teachers of the above-mentioned higher education institution participated in the project and conducted this study.

#### 2.2.1. Preparatory Phase of THR Lessons

Two weeks before the start of the THR lessons, the instructors conducted training for nursing students on the specifics of THR and working with horses. The training was conducted in four phases: (1) theoretical and practical education of students on specific procedures with horses, (2) teaching students riding skills, (3) educating students on the natural bioenergy of manure, and (4) developing a socially beneficial learning plan through THR activities. Nursing students and teachers spent a week before THR sessions educating parents about the 14 components of basic life needs according to Henderson.

#### 2.2.2. Implementation Phase of THR Lessons

Over six months, each child participated in a THR lesson once per week (24 lessons in total). Each lesson lasted for 25 min with two students, a THR instructor, and a minimum of one parent working with the child riding a horse. Depending on the type and severity of the child’s disorder, the appropriate horse was selected for each individual child. According to the individual program, during THR sessions the children performed leaning forward exercises to touch the horse’s neck, leaning back to touch his rump, and twisting left and right on the horseback while the horse was in a slow, steady walk [22]. Nursing students evaluated and noted the child’s appearance, general condition, behavior, mood and safety before, during and after each THR lesson.

### 2.3. Participants

The study involved parents of 13 children with disabilities, aged 4 to 14 (Table 2). All participants lived in the area where the study was conducted. Purposive sampling, based on intended research outcomes, was performed according to the defined criteria [23]. The sample size was further determined based on informational needs [24]. To be included in the study, the parents had to meet the following criteria: (1) membership in the Association for Parents of Children with Disabilities involved in the project, (2) a parent (or legal guardian) of a child or children with disability or disabilities (aged 4 to 18), (3) voluntary participation in the study, (4) full collaboration with researchers, and (5) continuous participation of the child in all 24 THR lessons. None of the children included in this study had previously been enrolled in THR programs.

Five families were not included in the study because three of them did not want to participate in the study, and two children did not regularly attend THR lessons.

### 2.4. Data Collection

During the six months of THR, parents observed their child’s basic life needs and recorded noticeable changes on paper forms provided by the researcher. Due to the anonymity of the participants, the forms included an encrypted ID code (a combination of letters and numbers). Following Bengtsson [24], non-suggestible, open-ended written questions in the paper form were used to encourage parents to respond. Parents also stated that for them a written form of expression is simpler than oral because it does not cause tension and anxiety, offers longer response times and provides less risk of forgetting and mistakes. Open-ended written questions provide parents with an extra-secure flow of thought, free writing, and a detailed description of changes in their children, which cannot be achieved with structured questions and a list of offered, preformulated answers [4,24]. The parents were asked to (1) describe any changes in the basic life needs of their child that was noticed in six months of THR lessons and (2) explain why these changes occurred in your child’s basic life needs.

Parents were asked to write their reports honestly, with a detailed description of every change in their child’s basic life needs, without restrictions on the amount of written text. Parents were further advised to base their reports on facts consistent with Henderson’s 14 components (Table 1). The parents submitted the final written reports to the researchers after six months (after 24 THR lessons).

The triangulation method was applied to eliminate biases [24]. To minimize parental bias and the phenomenon of “cognitive impenetrability of visual perception” when opinions, emotions, and expectations affect the content of parents’ vision [15,25,26], estimates were longitudinal with reports from both parents. Following the remarks of Elo and Kyngäs [27], all parents received the final coded data to verify responses. For the additional confirmation of parents’ reports, the assessments of professionals (psychologists, speech therapists, physical therapists, occupational therapists and THR instructors), who observed the children during regular diagnostic and therapeutic activities (e.g., sensory integration, neurofeedback, kinesiotherapy, etc.), were also analyzed. They wrote their observations on a structured form that included six areas of the child’s functioning (collaboration, communication, attention and mental concentration, motor skills, spatial and temporal orientation, and emotion control), and submitted the final reports to the researchers after 24 THR lessons [28].

### 2.5. Data Analysis

In this qualitative study, a deductive approach was employed using content analysis [23,26]. Content analysis can be used on all types of written texts no matter how the data is collected and helps a deeper understanding of human perceptions [27]. Since the study is based on the previously developed Henderson’s theory, a deductive approach has been applied in data analysis. This approach is often used in cases where the researcher wishes to retest existing data in a new context or test previously developed theories, concepts, models or hypotheses [27]. According to the data collection method (open-ended written questions), the manifest approach was used [29]. Hence, data collection method directly affected the depth of the analysis in this study. Considering the research questions and theoretical study framework, three researchers (the authors of this paper with experience in qualitative data processing) deductively developed a structured categorization matrix and formed categories [27]. Subsequently, they independently reviewed and analyzed the data, and then coded the data according to the identified categories in the categorization matrix [24]. Since the matrix is structured, the researchers extracted from the data only those aspects that fit the matrix of analysis [27]. This procedure has been used as a form of triangulation because there is always a risk that different researchers may draw different conclusions from different data [24]. Therefore, at least two researchers need to perform data analysis separately [24]. Upon finishing individual reviews and data coding, we completed a common analysis and final organization of the data and reached a final consensus [26].

### 2.6. Ethical Considerations

All participants were informed of the purpose and other details of the study. Children were presented with information tailored according to their age, intellectual, and cognitive characteristics. Participation in the study was voluntary and the participants had the right to withdraw from the study without any consequences. Parents voluntarily signed the informed consent for their own and their child’s participation in the study. The anonymity of the participants was guaranteed, and there was no possibility to determine their identity from the responses. To ensure confidentiality, participants received an encrypted code. Only researchers had access to research data. The study was conducted in accordance with the Declaration of Helsinki, and approved by the Committee of the Faculty of Dental Medicine and Health Osijek (04-2-1-02-0186).

## 3. Results

The deductively formed categorization matrix included 16 categories (CAT) within two generic categories: (1) parental perception of changes in their child’s basic life needs and (2) parental perception of factors that caused changes in their child’s basic life needs. The final organization of data defined a total of 40 codes within 13 CATs (Table 3).

### 3.1. Parental Perception of Changes in Their Child’s Basic Life Needs

**Figure 1:**
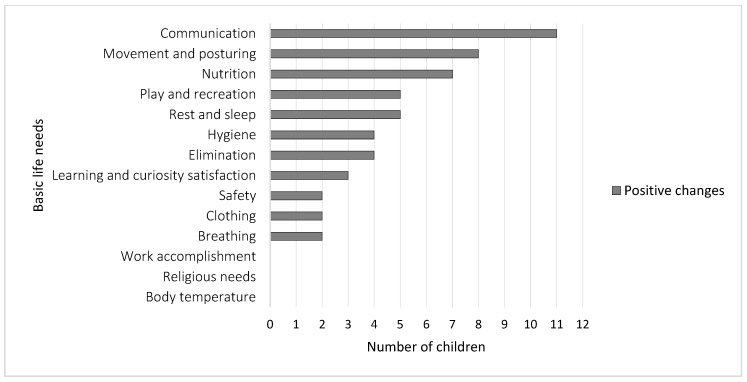
Incidence of positive changes in children’s basic life needs.

Qualitative analysis of parents’ reports indicated that parents of 11 children recorded only positive changes in their children’s basic life needs, whereas parents of two children (C10 and C13) did not record any changes (Table 3). Furthermore, benefits of THR were determined in 11 of the 14 need theory components (Figure 1).

The results of the analysis of the parents’ reports are provided below according to the number of identified codes in each CAT.

Most (seven) codes were identified in CAT “relationships and child’s communication with other people (emotions, fears, moods, opinions)”. For example, according to parents’ reports, a better mood following THR lessons was observed in ten children, a higher level of calmness and patience in five children, and more frequent communication with other people in four of them. For example, the parents affirmed:

*“Our child is content, happier, and laughs more. On every occasion, he is very pleased to meet the horse and THR instructors”* (P9) and *“Meeting new people is no longer a problem for our child. The child communicates with other people more often and with better quality. He used to say only 3 to 5 words, and now he pronounces up to 10 words in continuity”* (P11).

A total of five codes are associated with the “movement and posturing” CAT. Parents of eight children reported improved mobility of their wrist and leg joints after THR lessons, and four children had better movement coordination and more autonomy in moving, e.g.,

*“Our child has better movement control. He takes items more firmly and securely. For example, he no longer drops a pen or a glass so often”* (P6).

In two cases, parents reported better resilience in their child:

*“When we are walking, the child runs and jumps much more and gets less tired than before”* (P4).

Four codes are identified in two CATs: “rest and sleep” and “elimination of body wastes”. Parents have reported easier bedtime and more peaceful sleep in four children, e.g.,

*“My husband and I no longer have the usual long-lasting evening rituals of putting our child to sleep. Our child has a much peaceful sleep and does not wake up often during the night”* (P3).

In four children, parents reported more frequent and copious stool, and one child had better control of urinary output, e.g.,

*“For the past few years, our child has had constipation problems. She defecated every fourth to fifth day, with intense strain and frequent abdominal pain. The stool was often of very hard consistency. Now she defecates every other day, without accompanying pain”* (P12);

*“The irregular stool in our child now belongs to the past, although the child still uses medications that cause constipation as a side effect. This change in our child is precious”* (P3); and *“During the day, our child announces the urge to go to the toilet, so we use the usual absorbent products only during the night”* (P6).

The three codes are identified in three CATs: “food and fluid intake”, “breathing”, “play and recreation”. In six children, parents reported better appetite and more fluid intake, and in two children consuming more diverse kinds of food, e.g.,

*“Ever since our child has been included in THR activities, he has a much better appetite and drinks twice as much fluid than before”* (P5) and *“The child now consumes 3–4 meals a day. Previously, he rejected food and ate twice a day at most”* (P1).

According to parents’ reports, two children found it easier to breathe during the day, without rapid breathing at rest, whereas in one child, parents reported that he breaths more peacefully in his sleep:

*“At night, the child no longer suffers from episodes of rapid breathing and sleeps much calmer”* (P2).

In five children, parents reported their greater interest in playing with others, and two children became more interested in spending time in nature, e.g.,

*“Going out in nature and playing with friends became important to our child. Previously, he had separation anxieties and avoided the company of other kids”* (P7).

Parents also said that two children reduced cell-phone use for entertainment purposes:

*“Finally! The child is separated from his cell-phone and goes to the park with his friends”* (P5).

Two codes are identified in two CATs: “maintaining personal hygiene” and “learning and curiosity satisfaction”. For example, parents reported more frequent hygiene practices in four children, e.g.,

*“Our child always had a problem with washing his hands, but now, without us reminding him, the child washes hands all on his own”* (P9).

In three children, parents reported more interest in learning and easier understanding of school material, e.g.,

*“Our child will have learning difficulties for their lifetime, but we recognized a greater desire for reading content about animals”* (P2).

One code is identified in two CATs: “clothing” and “safety”. In the case of two children, parents identified more independence in dressing as well as desire for wearing protective gear before horseback riding, e.g.,

*“It is amazing that the child now puts both socks on independently, which was very problematic earlier”* (P6);

*“The child always asks for protective equipment before horseback riding. Sometimes she asks for a headgear already in the car, on the way to the Equestrian Club”* (P8).

In three deductively formed CATs (“body temperature”, “religious needs”, and “work accomplishment”), no changes were noticed by the parents.

An analysis of the assessments submitted by professionals indicates the specific progress in children with disabilities in all six observed areas, which further supports conclusions drawn from parents’ reports (Table 4).

### 3.2. Parental Perception of Factors that Caused Changes in Their Child’s Basic Life Needs

In parental reports, there are noticeable differences in perception of the factors that have caused positive changes in their children. In case of three children, the parents described positive changes but felt that THR was not the only contributing factor (Table 3). Additional important factors, in their opinion, were the child’s growth and development, the impact of other forms of therapy, medication, and work with children (CAT “positive changes due to therapeutic and situational factors”), e.g.,

*“There are obvious positive changes, but I think they are a result of several influencing factors, not just THR, for example, the impact of child’s development and the medication he uses daily”* (P1);

*“Simultaneously, our child attends neurofeedback and other forms of therapy, and we also work with the child every day. I do not believe that THR was crucial, but the improvements are a fact”* (P4).

In contrast, parents of eight children emphasize the direct connection between THR lessons and positive changes in their children. They identified the child’s contact with the horse’s body, the movements of the horse, and the child’s contact with different people, sounds and sensory stimuli during THR activity as essential (CAT “positive changes due to therapeutic horseback riding”), (Table 3), e.g.,

*“I think my child’s contact with the horse is a key factor in all the improvements. This is indisputable. We had previously and unsuccessfully tried with various other therapies and failed. THR has improved our child’s quality of life”* (P3).

## 4. Discussion

According to research questions, our aims were (1) to describe parental perceptions of changes in the basic life needs of their children with disabilities after THR lessons and (2) to analyze parental explanations of the factors that caused noticeable changes in their children.

### 4.1. Parental Perception of Changes in Their Child’s Basic Life Needs

According to parents’ reports, only positive changes in the basic life needs were observed in as many as 11 children after THR lessons, whereas parents of two children did not record any changes.

The most improvements were reported in CAT “child’s relationships and communication with other people (emotions, fears, moods, opinions)”, which is fully supported by the results of other international studies [1,4,8,9,11,12,30]. So, according to parents’ reports, four children communicated with other people more often after THR. The same benefits of THR in children with ASD were described by Gabriels et al. [8]. Improvements in social interaction in children with disabilities are often the result of the engagement of everyone involved in THR, their communication with the child, and encouraging the child to communicate with the horse [9]. Parents in this study noted that the collaboration of children with adults and horses reduced children’s fears of strangers and animals, which is supported by the results of an Australian study in which children with cerebral palsy experienced reduced fear of strangers, horses, and heights after THR sessions [18]. Horse movements had a positive effect on the mood, calmness, and patience of children involved in this study, decreasing the occurrence of their emotional outbursts and aggression toward other people. This calming effect of THR through synchronized horse movements has been also reported in two studies [9,11]. During THR, children’s emotional self-control also improves because they must adjust their behavior while horseback riding so they could better interact with the animal [4]. We found that four children developed significantly higher levels of self-esteem, which is consistent with parents’ reports analyzed in other studies [4,7,31]. Specifically, the relevant psychological research on the impact of activity and therapy with horses on the self-esteem of children and adolescents shows different and opposite results, indicating the need for further research [32].

Eight children involved in our study experienced improvements in CAT “movement and posturing”. Positive changes included better posture, balance, mobility of the limb joints, and coordination of movements, and more independence and confidence while walking, as well as better physical fitness, which is in accordance with the results in other studies [4,33,34]. For example, in two South African studies [4,33], parents of children with diverse disabilities reported their better posture, gait, and balance, whereas Land et al. described improvements in postural control of children with disabilities after THR [34]. Three-dimensional rhythmic horse movements encourage the child to constantly adjust to changes in speed and direction, which improves the child’s posture, balance, and movement coordination [10]. A U.S. study conducted in 2010 showed that parents of disabled children noticed improvements in their coarse and fine motor skills, increased flexibility, and better hand coordination after THR [1]. Parents in our study reported higher levels of autonomy and child confidence while moving, and improved physical condition and fitness, which is supported by the results of other studies [35,36,37] that described improvements in walking, running, and jumping of children with cerebral palsy.

Four parents reported that their children fell asleep more easily after THR, and slept longer and more peacefully (CAT “rest and sleep”). Children with disabilities often have trouble sleeping [8,37,38]; however, a review of the relevant literature did not provide a study describing parental reports of the specific effects of THR on the quality of sleep in children with disabilities. Boyd and le Roux [4] described the immediate calming effect of THR as children fell asleep on horseback while riding. Horse body heat and horse movements are known to further calm the children [8]. Those with ASD often use different medications [39] due to sleeping problems, hyperactivity, depression, or aggression. As a child with ASD involved in this study reported a better quality of sleep after THR, this could provide incentive for future research.

The results of this study showed that four children experienced significant improvements in CAT “elimination of body wastes” after THR. According to parents’ reports, children defecated more often, they had a more copious stool, and announced their urge to go to the toilet. One child had better control of his urine output and went to the toilet on his own. Unfortunately, problems with constipation and painful defecation are common in children with disabilities [40] as well as urinary retention problems [41]. Horse rhythmic movements are known to mobilize the child’s pelvic girdle and muscles, thus stimulating intestinal peristalsis and elimination, which is vital for a child’s well-being [42]. We found no relevant study in which parents of children with disabilities reported specific effects of THR on the elimination of body wastes. Therefore, these benefits could also incentivize further research.

According to parents’ reports, six children involved in this study had a better appetite after THR and consumed more fluid (CAT “food and fluid intake”). Children with disabilities, due to frequent disorders of sensory experiences (especially smell and taste), or symptoms such as loss of appetite, have problems with food and fluid intake [9]. In this study, three children had sensory integration disorder. Their parents reported that their children had always avoided certain odors and flavors, as well as meals, but after THR, children became more tolerant of various sensory stimuli, which improved their appetite. These sensory benefits of THR were also recognized in other studies [7,8].

The parents of two children in this study reported that their children were able to breathe more peacefully during the day and night due to THR and regular therapy, without the usual episodes of rapid breathing (CAT “breathing: respiratory and circulatory status”). THR is known to positively influence breathing and circulation stimuli in children with disabilities [33]. Breathing relief, according to parents’ reports, also had a secondary positive effect on the quality of sleep, movement, physical fitness, and mental concentration in their children.

According to parents’ reports, five children showed more interest in playing with others after THR and two children reduced their use of a cell-phone for entertainment purposes (CAT “play and recreation”). The observed increase of social integration corresponds with the findings of other similar studies [4,9,10,16]. Many factors, such as the natural environment, the experience a horse provides to the child, and the child’s exposure to many interactions with instructors, therapists, volunteers, as well as with other children, contribute significantly to the child’s integration into society [7]. Horseback riding provides a positive encouragement for the child, leading to increased motivation and social interaction [4].

Four children involved in this study implemented hygiene practices more often and with increased independence after THR (CAT “maintaining personal hygiene”). A literature search showed no available relevant study describing similar benefits of THR, although problems with hygiene practices in children with disabilities are no exception [42]. For example, children with ASD, due to deficits in sensory processing, have various behavioral problems, and among them avoiding hygiene procedures for washing face, hair, or teeth [42].

Parents of three children with learning disabilities reported their increased interest in learning after THR and their easier understanding of school material (CAT “learning and satisfaction of curiosity”). This result supports Stickney [1], who concluded that THR has a positive effect on the schoolwork of children with ASD. The child must listen to the directions of the THR instructor while focusing on horseback riding, which has a positive effect on motor planning, general focus, mental concentration, and problem-solving abilities [1,11].

In two children lacking normal physiological development, parents reported higher levels of independence in dressing themselves (CAT “clothing”), fully supporting the study in which parents of children with ASD reported their improved motor control, flexibility, and arm coordination, especially when they were dressing themselves [1].

In the case of two children, parents noted their readiness to use protective gear before horseback riding (CAT “safety”). THR is known to stimulate the development of a child’s own body and ego awareness, empowering children to take risks, increasing their self-discipline and emotional control [10]. So, this is a crucial benefit because children become more aware of their body’s vulnerabilities and potential dangers, as well as the importance of adequate protection.

Parents of two children (C10, C13) involved in our study did not record any changes, which is a result supported by other studies [18,22,33,43]. The impact of THR on an individual child with disabilities depends on the type and severity of disability or disabilities [44] as well as on the individual specificities of the child’s organism [43]. A child (C10) whose parents have not noticed any changes suffers from DiGeorge syndrome with complex pathology and severe symptomatology. Furthermore, the impact of THR on a child also depends on the total duration and number of THR sessions, the duration of each THR lesson [33,45], the type of THR program (individual or group therapy [4], the application of different measuring instruments [43], the duration of observing the changes in a child [44], and subjective factors of parents as assessors [26]. The parents’ assessments in this study were supported by reports submitted by professionals, indicating the specific physiological and psychological progress of children (Table 4). Thus, professional evaluation reduced the risk of possible biases in parental assessments and improved the validity of the results presented in this study [15].

### 4.2. Parental Perception of Factors that Caused Changes in Their Child’s Basic Life Needs

The parents involved in the present study largely considered THR to be a major factor producing positive changes in their child’s basic life needs. However, parents of three children stated that the improvements are, rather, a result of a combination of THR and the effect of specific therapies (e.g., sensory integration, neurofeedback, kinesiotherapy), as well as child’s normal psychomotor development (CAT “positive changes due to therapeutic and situational factors”). These results are fully supported by Boyd and le Roux’s [4] study in which only a minority of parents believed that a combination of different factors and circumstances helped their children. Boyd and le Roux stated that these parental perceptions are supported to a certain extent, given that important physical, emotional, social, and cognitive changes occur in children from the age of six until the end of adolescence [4,46].

Eight children’s parents were convinced that THR was responsible for all the improvements noticeable in their children. As key factors, they indicated direct contact of the child with the horse’s body and its movements, interaction of the child with different people, and exposure to different sounds and sensory stimuli during THR lessons (CAT “positive changes due to therapeutic horseback riding”). These results are supported by studies that confirmed the therapeutic effect of synchronized horse movements [1,9], horse’s body temperature [47], communication with instructors and other THR participants, and their encouragement of the child to communicate with the horse [9]. The parents confirmed that their children have been subject to other forms of therapy for a long time, but lacked such evident positive effects on their child’s basic life needs. For example, Ward et al. confirmed that the positive therapeutic effects of THR in children with ASD were not sustainable during the six-week break from THR, but benefits reoccurred when THR began [12].

### 4.3. Limitations and Recommendations for Future Research

In this six-month study, participants included only parents whose children with disabilities were involved in a regional THR program. Therefore, the sample is not large enough to generalize the results to all the children with disabilities involved in THR programs throughout the Republic of Croatia. In the future, a study of more than six months duration should be planned and, if possible, more respondents from more than one region in Croatia should be included. Furthermore, future research could include differentiation of various possible factors such as diagnostic groups of children with disabilities, age and other factors. Furthermore, it would be useful to apply a combined research approach and, with quantitative confirmation of the outcomes, contribute to a higher level of objectivity to better understand the specific effects of THR on children with disabilities. Furthermore, future research of parental perceptions should be based on holistic and individualized assessments of children with disabilities in terms of their basic life needs and their independence in performing daily life activities.

### 4.4. Usefulness and Applicability of Study Results

The results of this study indicate the usefulness and applicability of Henderson’s human needs model in working with children with disabilities. The model can be used by parents and professionals for the purpose of ongoing observation and evaluation of the condition and possible changes in the basic life needs of children with disabilities undergoing THR and other forms of therapy. Furthermore, Henderson’s model can be of significant help in the design and revision of individualized THR programs and part of common practice when working with children with disabilities. This study further justifies the usefulness of THR in the prevention and treatment of various symptoms and side-effects in children with disabilities (e.g., heavy breathing, impaired communication, loss of appetite, impaired sensation, urinary and constipation disorder, impaired mobility, sleep disturbance, etc.). Preventing and alleviating these symptoms and side-effects can advance the treatment and rehabilitation process and significantly improve the quality of life of children with disabilities.

## 5. Conclusions

In this study, we described parental perceptions of the effects of six-month THR sessions on the basic life needs of children with disabilities from the perspective of Henderson’s need theory. The results indicated that parents report only positive changes in basic life needs and an increased level of independence in 11 children with disabilities. These results were largely supported by the assessments by professionals involved in the diagnostic/therapeutic work with children. Most parents stated that THR was responsible for all the physical, psychosocial, and cognitive benefits noticeable in their children. Furthermore, according to parents, all of the above ultimately leads to a generally improved their children’s quality of life. The results are supported by relevant literature. To the best our knowledge, this is the first qualitative study to describe parental perceptions of the effects of THR on children with disabilities applying Virginia Henderson’s need theory. We also described the specific impact of THR on, for example, children’s sleep and rest, personal hygiene maintenance, and elimination of stool and urine, which has not yet been explored in detail in previous studies describing parental perceptions of the impact of THR on children with disabilities. Therefore, we expand upon the existing global scope of knowledge about THR and contribute to better understanding of how THR affects the basic life needs of children with disabilities. Ultimately, the findings can help parents of children with disabilities enrolled in THR programs, therapists, and THR instructors in planning and implementing a holistic approach for assessing and satisfying the basic life needs of children with disabilities, from the aspect of Henderson’s need theory.

## Figures and Tables

**Table 1:** Description of the components according to Henderson’s theory.

Component	Aspect of Component	Description
1. Breathing	Physiological	Breathe normally
2. Nutrition	Physiological	Eat and drink adequately
3. Elimination	Physiological	Eliminate body wastes
4. Movement and posturing	Physiological	Move and maintain desirable postures
5. Rest and sleep	Physiological	Rest and sleep
6. Select suitable clothes	Physiological	Select suitable clothes
7. Body temperature	Physiological	Maintain normal body temperature
8. Hygiene	Physiological	Keep the body clean and well-groomed
9. Safety	Physiological	Avoid dangers in the environment and avoid injuring others
10. Communication	Psychological	Communicate with others in expressing emotions, moods, opinions
11. Religious needs	Spiritual and moral	Worship according to one’s faith
12. Work accomplishment	Sociological	Work in such a way that there is a sense of accomplishment
13 Play and recreation	Sociological	Play or participate in various forms of recreation
14. Learning and curiosity satisfaction	Psychological	Learn, discover, or satisfy the curiosity

**Table 2:** General data on the participants.

Parents	Child	Child’s Disabilities
P1	C1	Intellectual disabilities Speech and language impairment in voice communication, and learning disabilities
P2	C2	Physical activity and attention disorders Speech and language impairment in voice communication, and learning disabilities Intellectual disabilities
P3	C3	Autism spectrum disorder Speech and language impairment in voice communication, and learning disabilities Intellectual disabilities Sensory integration disorder
P4	C4	Intellectual disabilities Visual impairment Sensory integration disorder
P5	C5	Intellectual disabilities
P6	C6	Lack of expected normal physiological development Speech and language impairment in voice communication, and learning disabilities Intellectual disabilities
P7	C7	Autism spectrum disorder Speech and language impairment in voice communication, and learning disabilities Behavioural disorders and mental health impairment Sensory integration disorder
P8	C8	Down syndrome Speech and language impairment in voice communication, and learning disabilities Psychophysical development retardation
P9	C9	Intellectual disabilities Psychophysical development retardation Behavioural disorders and mental health impairment
P10	C10	DiGeorge syndrome Intellectual disabilities
P11	C11	Cerebral palsy Intellectual disabilities Psychophysical development retardation
P12	C12	Cerebral palsy Psychophysical development retardation
P13	C13	Hyperkinetic disorders Speech and language impairment in voice communication Psychophysical development retardation

**Table 3:** The process of data abstraction contained in parents’ reports.

GC	Category	Code (Changes in Children Basic Life Needs)	Parents’ Reports (P1–P13)												
			P1	P2	P3	P4	P5	P6	P7	P8	P9	P10	P11	P12	P13
**GC 1**	Breathing	Easier breathing during the day		X		X									
		Breathing is not accelerated at rest		X		X									
		Breathing more peacefully during sleep				X									
	Food and fluid intake	Better appetite	X		X		X	X			X		X		
		Ingestion of more fluid	X		X		X	X			X		X		
		Consuming various types of food							X		X				
	Elimination of body wastes	More frequent and copious stool			X		X	X						X	
		Announcing the urge to go to the toilet			X			X							
		Better control of urine output			X										
		Going to the toilet alone												X	
	Movement and posturing	More flexible wrist and leg joints		X	X	X	X	X	X	X			X		
		Better coordination of movements		X		X		X					X		
		Better physical fitness		X		X									
		Better body posture and balance when walking		X		X		X					X		
		More independence in moving		X		X		X					X		
	Rest and sleep	Easier bedtime	X	X	X					X					
		A more peaceful dream	X	X	X					X					
		Longer sleep	X	X	X					X					
		Going to sleep alone		X					X						
	Clothing	More independence in clothing						X					X		
	Maintaining personal hygiene	More frequent maintenance of personal hygiene			X	X					X			X	
		More independence in maintaining personal hygiene			X	X					X			X	
	Safety	Desire to wear protective gear before horseback riding							X	X					
	Relationships and child’s communication with other people (emotions, fears, moods, opinions)	Better mood throughout the day		X	X	X	X	X	X	X	X		X	X	
		A higher level of calmness and patience					X		X	X	X			X	
		Higher self-esteem					X		X	X				X	
		More audible speech	X		X										
		Establishing more communication with other people	X		X				X				X		
		Lower levels of fear of strangers and animals					X		X	X				X	
		Less aggression towards people									X				
	Play and recreation	More interest in playing and recreation	X				X		X	X	X				
		Desire to stay in nature							X	X					
		Reduced use of cell-phone for entertainment	X				X								
	Learning and curiosity satisfaction	More interest in learning	X	X							X				
		Easier acquisition of school material	X	X							X				
**GC 2**	Positive changes due to therapeutic and situational factors	The child’s growth and development	X												
		Effect of other forms of therapeutic work and medication	X			X								X	
	Positive changes due to therapeutic horseback riding	Child’s contact with the horse’s body		X	X		X	X	X	X	X		X		
		Horse movements		X	X		X								
		Contact with people, exposure to sounds and sensory stimuli		X	X		X								

GC 1 = Generic category 1 (Parental perception of changes in their child’s basic life needs); GC 2 = Generic category 2 (Parental perception of factors that caused changes in their child’s basic life needs); “X” = Code (positive change) identified in parents’ reports.

**Table 4:** Reports of professionals on specific changes in children with disabilities.

Aspect of Assessment	Professional Reports	Children (C1–C13)
Collaboration	Better collaboration; better execution of verbal instructions and tasks; better acceptance of therapy.	C5, C7, C8
Communication	Establishing communication independently; better quality of communication; more frequently asked questions.	C1, C3, C7
Attention and mental concentration	Improved focus on activities; longer attention; better mental concentration.	C5, C8, C9, C12
Motor skills	Better posture; better movement coordination and balance in standing and walking.	C2, C4, C6, C8, C11
Spatial and temporal orientation	Improved spatial orientation in moving.	C2
Emotional control	Better control of emotions.	C5, C7, C9
